# *EGFR* and *TP53* mutations refine prognostic stratification of *IDH*-wildtype astrocytomas, not elsewhere classified

**DOI:** 10.1186/s40478-026-02397-6

**Published:** 2026-08-03

**Authors:** Nayuta Higa, Toshiaki Akahane, Seiya Yokoyama, Taiji Hamada, Hajime Yonezawa, Ryutaro Makino, Tomoko Takajo, Mari Kirishima, Ikumi Kitazono, Yutaka Fujioka, Ryusuke Hatae, Daisuke Kuga, Shohei Nagasaka, Kohei Suzuki, Junkoh Yamamoto, Koji Yoshimoto, Akihide Tanimoto, Ryosuke Hanaya

**Affiliations:** 1 Department of Neurosurgery, Kagoshima University Graduate School of Medical and Dental Sciences, 8-35-1 Sakuragaoka, Kagoshima City, 890-8520 Japan; 2 Department of Pathology, Kagoshima University Graduate School of Medical and Dental Sciences, 8-35-1 Sakuragaoka, Kagoshima City, 890-8544 Japan; 3 Center for Human Genome and Gene Analysis, Kagoshima University Hospital, 8-35-1 Sakuragaoka, Kagoshima City, 890-8544 Japan; 4 Department of Neurosurgery, Graduate School of Medical Sciences, Kyushu University, 3-1-1 Maidashi, Higashi-ku, Fukuoka, 812-8582 Japan; 5 Department of Neurosurgery, University of Occupational and Environmental Health, Yahatanishi-ku, Kitakyushu, 807-8555 Japan

**Keywords:** *IDH*-wildtype astrocytoma, Not elsewhere classified, *EGFR*, *TP53*, DNA methylation profiling, Prognostic stratification, Glioblastoma

## Abstract

*IDH*-wildtype astrocytomas of histological grade 2 or 3 without the WHO 2021 molecular criteria for glioblastoma—*TERT* promoter mutation, *EGFR* amplification, or combined whole-chromosome 7 gain and 10 loss (+ 7/– 10)—are designated “Not Elsewhere Classified” (NEC), a residual category with clinical behavior ranging from indolent disease to courses clinically indistinguishable from glioblastoma (GBM). However, reliable molecular markers capable of resolving this heterogeneity have not been established. We retrospectively profiled a multi-institutional cohort comprising 38 adult *IDH*-wildtype astrocytoma NEC cases and 429 GBM controls using a bimodal DNA/RNA next-generation sequencing panel and, in 30 NEC cases, genome-wide DNA methylation profiling. NEC tumors were markedly heterogeneous: 21 of the 30 methylation profiles (70%) failed to match any reference class family, and the remaining nine were distributed across six categories, with Uniform Manifold Approximation and Projection (UMAP) showing scattered embedding adjacent to multiple high- and low-grade reference clusters, rather than a coherent NEC cluster. Against this heterogeneous background, two point mutations emerged as candidate markers for aggressive disease: *EGFR* mutations (3/38, 7.9%), all clustered in the extracellular domain, and *TP53* mutations (8/38, 21.1%), predominantly in the DNA-binding domain. Both retained independent prognostic significance in Firth-penalized multivariate Cox regression (*EGFR*: HR 1.61, 95% CI 1.02–2.57, *P* = 0.028; *TP53*: HR 1.55, 95% CI 1.00–2.42, *P* = 0.041). NEC patients harboring *EGFR* and/or *TP53* mutations showed an overall survival curve closely paralleling that of GBM (*P* = 0.9605) and significantly diverged from *EGFR*/*TP53*-wildtype NEC (*P* = 0.0011). Thus, despite the marked genomic and epigenomic heterogeneity within NEC, *EGFR* and *TP53* point mutations may identify a high-risk NEC subgroup. This stratifier is deployable with routine targeted sequencing panels already widely used in diagnostic practice, and provides a practical refinement complementary to the cIMPACT-NOW Update 11 framework, pending prospective validation.

**Supplementary Information:**

The online version contains supplementary material available at https://doi.org/10.1186/s40478-026-02397-6.

## Introduction

Adult-type diffuse gliomas have undergone profound reclassification following the adoption of molecularly defined entities. The 2021 World Health Organization (WHO) Classification of Central Nervous System Tumors formalized a molecular diagnostic framework in which *IDH*-wildtype astrocytomas occupy a central diagnostic role [1]. Building upon cIMPACT-NOW Update 3, the WHO 2021 classification mandates that histological grade 2 or 3 *IDH*-wildtype astrocytomas harboring *TERT* promoter (*TERTp*) mutation, *EGFR* amplification, or combined whole-chromosome 7 gain and whole-chromosome 10 loss (+ 7/ − 10) be designated as “Glioblastoma, *IDH*-wildtype, CNS WHO grade 4,” even in the absence of histological hallmarks of glioblastoma [1, 2]. This molecular glioblastoma (mGBM) construct was introduced to reduce the risk that biologically aggressive tumors would be undertreated because histology alone would classify them as lower-grade [3–7]. It should be emphasized, however, that these molecular criteria were introduced as an early and explicitly provisional recommendation intended for iterative refinement rather than as fixed diagnostic thresholds [2]. The prognostic sufficiency of an isolated *TERTp* mutation, in particular, has since been questioned, as several series have found that histological grade 2–3 *IDH*-wildtype astrocytomas carrying an isolated *TERTp* mutation do not uniformly follow a glioblastoma-like course [6–10], although this point remains debated, with other work continuing to support *TERTp* mutation as a molecular criterion for grade 4 [11].

However, a clinically and biologically distinct subgroup remains outside both the histological and molecular definitions of glioblastoma. These tumors—*IDH*-wildtype astrocytomas of histological grade 2 or 3 that lack mGBM-defining molecular alterations—are designated as “Not Elsewhere Classified” (NEC) [12]. This category was originally proposed to acknowledge biologically uncertain tumors that resist confident assignment within the current taxonomy. As molecular profiling has matured, however, NEC has become less a uniform residual category than a heterogeneous group of tumors with markedly variable clinical outcomes. Some NEC tumors follow an indolent course consistent with lower-grade biology, whereas others show unexpected aggressiveness and rapid progression [11, 13]. This variability has direct clinical implications as reliable molecular markers capable of separating indolent from aggressive NEC tumors have not been established.

Notably, despite its clinical relevance, this residual category has received comparatively little dedicated attention in the successive frameworks that define it, remaining largely unaddressed in the cIMPACT-NOW updates and the WHO 2021 classification [1, 2, 14]. Its very existence has also been debated: some have argued that the diagnosis of grade 2–3 *IDH*-wildtype astrocytoma largely dissolves once comprehensive molecular testing is applied, with most cases reclassified as molecular glioblastoma [15], whereas others have provided evidence for a genuine, albeit rare, “true” lower-grade *IDH*-wildtype astrocytoma that follows a course distinct from glioblastoma [7, 16, 17]. Clarifying the biology of this contested group is therefore of considerable value, both to confirm that a subset of these tumors is genuinely indolent and to identify the minority that behave aggressively.

Among molecular alterations relevant to *IDH*-wildtype astrocytomas, *EGFR* is particularly important as it represents one of the most frequently altered oncogenes in glioblastoma, with amplification reported in approximately 40–60% of cases [18, 19]. Although the WHO 2021 classification incorporates *EGFR* amplification as a defining marker of mGBM, somatic *EGFR* point mutations represent a biologically and mechanistically distinct alteration class that remains outside the current diagnostic framework. Unlike amplification, activating *EGFR* point mutations may drive constitutive downstream signaling, potentially capturing an aggressive phenotype not covered by the current WHO 2021 criteria. Indeed, the biological consequences of *EGFR* alterations—encompassing activating extracellular-domain point mutations and the *EGFRvIII* rearrangement—have been characterized in detail in glioma and other cancers, where they can promote ligand-independent, constitutive signaling and extensive epigenome and transcriptional-network remodeling [20–23]. Their prognostic impact has been examined across both *IDH*-wildtype and *IDH*-mutant gliomas, including studies that directly compare amplification, *EGFRvIII*, and point mutations [17, 24–26], underscoring that *EGFR* activation can influence outcome through mechanisms not fully captured by amplification status alone. In parallel, *TP53*, the most frequently mutated tumor suppressor gene in human cancers, encodes a central regulator of genomic integrity, DNA damage response, apoptosis, and cell-cycle control [18, 27]. In adult-type diffuse gliomas, *TP53* mutations classically define *IDH*-mutant astrocytomas, in which they typically co-occur with *ATRX* inactivation [28]. In *IDH*-wildtype glioblastoma, *TP53* mutations are observed in a subset of tumors and may reflect impaired genomic surveillance and treatment resistance. However, whether *TP53* mutations carry comparable prognostic weight in *IDH*-wildtype astrocytomas—particularly within NEC, remains insufficiently examined. The molecular determinants of aggressive behavior in *IDH*-wildtype astrocytomas may extend beyond the currently recognized mGBM-defining alterations, and some NEC tumors may harbor as-yet-unrecognized molecular features, potentially including activating *EGFR* or inactivating *TP53* point mutations, which confer a clinical course equivalent to that of GBM.

Therefore, we focused on NEC tumors and performed comprehensive clinical, genomic, and genome-wide DNA methylation profiling, with particular attention to whether *EGFR* and *TP53* point mutations could serve as candidate prognostic markers for resolving NEC heterogeneity and refining risk stratification beyond the current WHO 2021 framework.

## Materials and methods

### Sample collection

This retrospective multi-institutional study was approved by the Institutional Review Board of Kagoshima University Hospital (approval nos. 180104 and 260,012) and was conducted in accordance with the Declaration of Helsinki. Written informed consent was obtained from patients enrolled from 2018 onward, and an opt-out approach was applied to cases prior to 2018. The inclusion criteria for *IDH*-wildtype astrocytoma, NEC, were as follows: (1) histological grade 2–3 IDH-wildtype diffuse astrocytomas confirmed by histopathology, (2) absence of *TERT* promoter mutation, *EGFR* gene amplification, or chromosome + 7/ − 10, (3) absence of *H3 K27* alteration or *H3 G34* mutation. From a multi-institutional glioma cohort, we collected 38 samples from adult patients who had been diagnosed with *IDH*-wildtype astrocytoma, NEC, between 2006 and 2025. For the survival analysis, 429 patients with *IDH*-wildtype GBM and 38 with *IDH*-wildtype astrocytoma, NEC, were used for comparison. The 429 *IDH*-wildtype GBM comprised 397 histological glioblastomas (CNS WHO grade 4) and 32 molecular glioblastomas (grade 2/3 histology meeting the WHO 2021 molecular criteria for glioblastoma; 31 carried a *TERT* promoter mutation and 12 an *EGFR* amplification).

### Histological examination

The resected tumor tissues were fixed in phosphate-buffered neutral 10% formalin and processed using routine formalin-fixed paraffin-embedded (FFPE) tissue preparation for histological, immunohistochemical, and genomic examinations. Representative FFPE sections and frozen tissues were subjected to genomic classification according to the WHO 2021 classification using a bimodal DNA/RNA panel by board-certified surgical and molecular pathologists (M.K. and A.T.).

### DNA and RNA extraction

DNA and RNA were extracted from FFPE Sects. (10-μm thickness) and fresh-frozen tissue samples using the AllPrep DNA/RNA Mini Kit (QIAGEN, Hilden, Germany) according to the manufacturer’s instructions. Separate sequencing libraries were prepared from the DNA and RNA fractions. For DNA library preparation, the DNA concentration was measured using the Qubit 3.0 Fluorometer with the dsDNA BR Assay Kit (Life Technologies, Carlsbad, CA, USA), and DNA quality was assessed using the QIAseq DNA QuantiMIZE Kit (QIAGEN). Only DNA samples with a quality check score < 0.04 were used for library preparation. DNA served as the template for PCR amplification and library preparation for next-generation sequencing (NGS) using the QIAseq Multimodal Panel (QIAGEN). For RNA library preparation, RNA was enzymatically polyadenylated at the 3′ end and reverse-transcribed into complementary DNA (cDNA). Template-switching oligonucleotides carrying universal primer sequences were incorporated at both ends of the cDNA, followed by target-enrichment PCR amplification using fusion gene-specific primers to prepare the libraries. This workflow reduces genomic DNA interference during amplification and enables detection of fusion transcripts involving previously uncharacterized partner genes.

### Next-generation sequencing analysis

NGS analysis was performed using a bimodal DNA/RNA panel [29]. Sequencing was performed on the NextSeq 1000 system (Illumina, San Diego, CA, USA). The amplicon sequences were aligned to the human reference genome GRCh37 (hg19) within the target region. Data were analyzed using the QIAGEN Web Portal service (https://www.qiagen.com/). A commercial bioinformatics analysis service (Mitsubishi Electric Software, Hyogo, Japan) was used to interpret sequencing results. Copy-number variations (CNVs) were calculated using the cloud analysis pipeline of the QIAGEN Web Portal and corrected for percentage tumor content. We defined amplification as > 5 copies, hemizygous deletion as 0.5 < x < 1 copies, and homozygous deletion (HD) as < 0.5 copies.

### DNA methylation profiling and uniform manifold approximation and projection (UMAP) analysis

DNA methylation profiling was performed using the Infinium MethylationEPIC v2.0 BeadChip Kit. Data were processed with Minfi (RRID: SCR_012830) [30], and differential methylation analysis was conducted with limma (RRID: SCR_010943). Central nervous system tumors were classified using the Molecular Neuropathology classifier (https://www.molecularneuropathology.org/mnp/). For dimensionality reduction, the genome-wide methylation data from our samples were integrated with reference data from GSE109381 (RRID: SCR_005012) [31] using Uniform Manifold Approximation and Projection (UMAP), as implemented in the umap R package. The UMAP embedding was computed on the 30 NEC samples together with 3,905 reference cases from NCBI GSE109381.

### Statistical analysis

Risk groups and clinicopathological characteristics were compared with the chi-square or Fisher’s exact test for categorical variables and the independent t-test for continuous variables (e.g., age, and Ki-67 index). OncoPrinter (cbioportal.org/oncoprinter), a tool in the cBioPortal for Cancer Genomics, was used to visualize and analyze the genomic data [32, 33], and survival analyses were performed using GraphPad Prism 11 software (MDF Co., Ltd.). To investigate the independent prognostic contribution of clinical and genetic alterations, we performed multivariate Cox proportional hazards regression analyses, which yielded hazard ratios (HRs) and 95% confidence intervals (CIs). Because of the limited cohort size and rarity of *EGFR* mutations, Firth-penalized Cox regression was used to reduce small-sample bias and address potential separation. To summarize the multiple correlated clinical variables in a single covariate, principal component analysis (PCA) was performed on centered and scaled variables (age, sex, histological grade, Ki-67 index, Karnofsky Performance Status, tumor location, dissemination status, and treatment-related variables), and the first principal component (PC1) was retained as a composite clinical covariate in the multivariate models. Overall survival (OS) was estimated using the Kaplan–Meier method and compared using the log-rank test. A *P*-value < 0.05 was considered statistically significant.

## Results

### Patient characteristics and genomic profile of NEC

We retrospectively analyzed 38 cases of *IDH*-wildtype astrocytoma classified as NEC according to the WHO 2021 criteria. The clinicopathological and molecular characteristics of the patients are summarized in Table 1. The cohort showed a slight male predominance (22 males [57.9%], 16 females [42.1%]), with a mean age of 59.2 ± 14.9 years (Table 1). A baseline Karnofsky Performance Status (KPS) score > 80 was recorded in 24 patients (63.2%), and gross resection ≥ 90% was achieved in 10 patients (26.3%) (Table 1). Histologically, 18 cases (47.4%) were grade 2 and 20 (52.6%) were grade 3, with a mean Ki-67 labeling index of 17.8 ± 17.3% (Table 1).

**Table 1:** Clinicopathological and molecular background of patients with NEC

Characteristics		NEC (n = 38)
Sex	Male	22 (57.9%)
	Female	16 (42.1%)
Age	Years	59.2 ± 14.9
KPS score	> 80 points	24 (63.2%)
	< 80 points	14 (36.8%)
Resection	≥ 90%	10 (26.3%)
	< 90%	28 (73.7%)
Histological grade	Grade 2	18 (47.4%)
	Grade 3	20 (52.6%)
Ki-67 (%)	17.8 ± 17.3	
*CDKN2A/B* HD	11 (28.9%)	
*NF1* loss and/or mut	10 (26.3%)	
*PTEN* loss and/or mut	10 (26.3%)	
*RB1* loss and/or mut	8 (21.1%)	
*TP53* mut	8 (21.1%)	
*PDGFRA* alterations	11 (28.9%)	
*EGFR* mut	3 (7.9%)	

KPS, Karnofsky Performance Status; mut, mutation; amp, amplification; HD, homozygous deletion

**Fig. 1:**
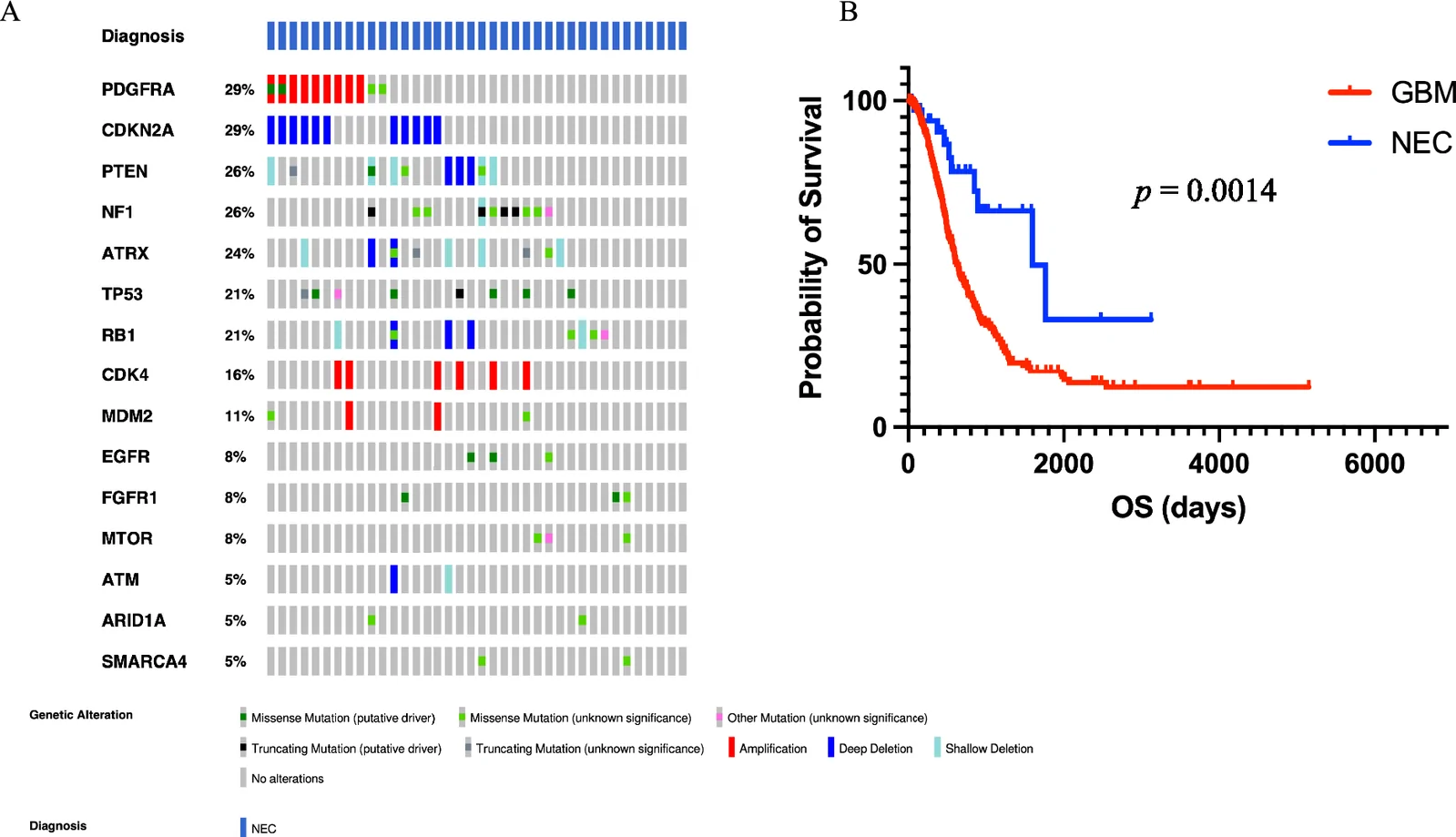
Genomic landscape and overall survival of NEC. **A** OncoPrint visualization of recurrent genetic alterations across the 38 NEC cases. **B** Kaplan–Meier curves of overall survival for GBM (n = 429) and NEC (n = 38). The *P*-value was derived from the log-rank test

Targeted NGS identified a diverse spectrum of genetic alterations across the NEC cohort (Fig. 1A). *PDGFRA* alterations and *CDKN2A/B* homozygous deletions (HD) were the most frequent alterations, each observed in 11 cases (28.9%), followed by *PTEN* loss and/or mutations in 10 cases (26.3%). *TP53* mutations were present in eight cases (21.1%), whereas *EGFR* mutations, which fell outside the WHO 2021 mGBM-defining alterations, were identified in three cases (7.9%) (Table 1 and Fig. 1A).

Kaplan–Meier analysis demonstrated that the OS of the NEC cohort was significantly better than that of the GBM cohort (*P* = 0.0014, Fig. 1B).

### Clinical and genetic prognostic factors in NEC

**Fig. 2:**
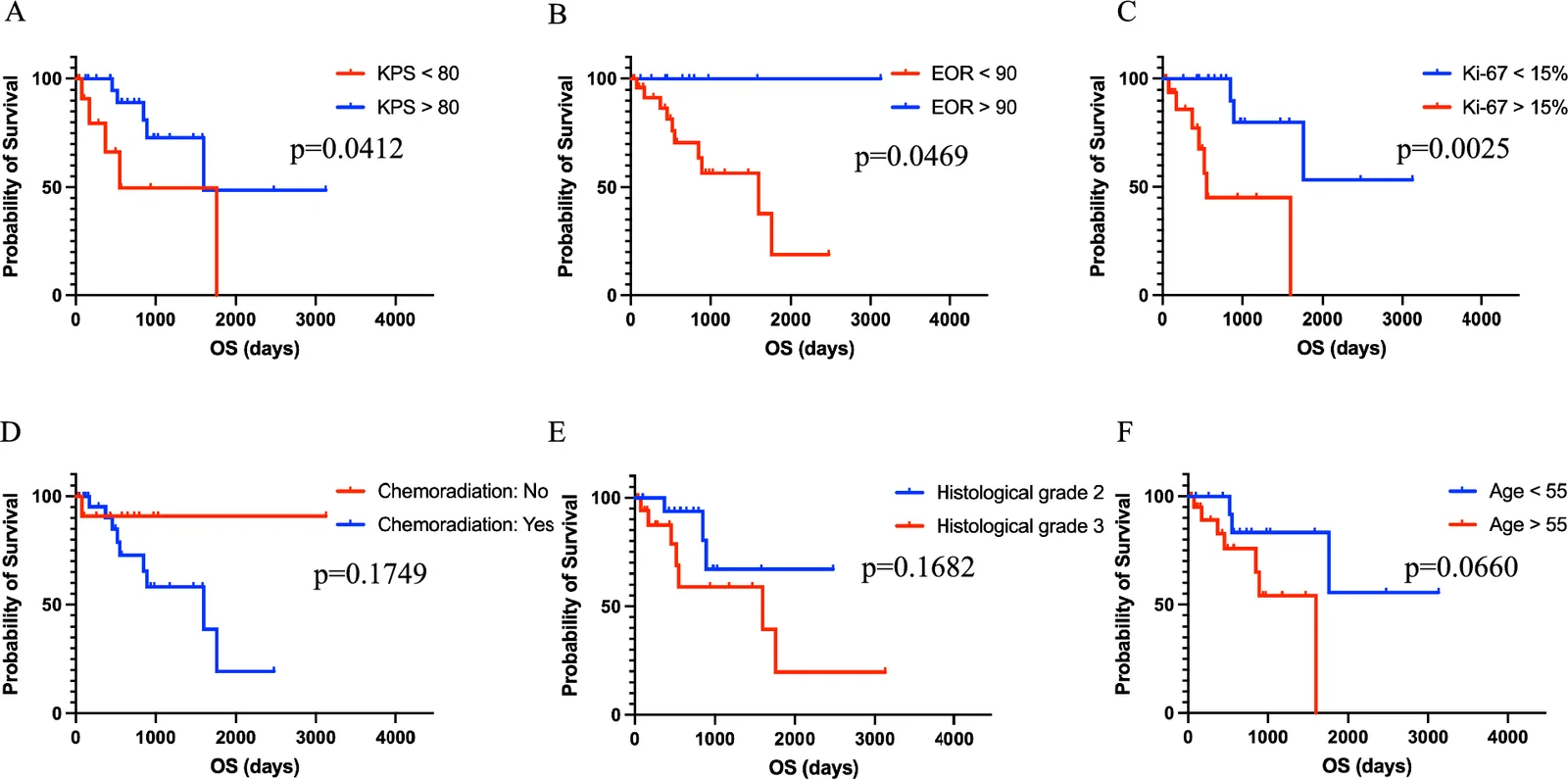
Impact of clinical factors on overall survival in NEC. Kaplan–Meier curves of overall survival stratified by **A** Karnofsky Performance Status, **B** extent of resection, **C** Ki-67 index, **D** chemoradiation, **E** histological grade, and **F** age. All *P*-values were derived from log-rank tests

To characterize the prognostic heterogeneity within NEC, we evaluated the association of clinical and genetic factors with OS in the NEC cohort (n = 38) (Fig. 2). Among the clinical factors, a preoperative KPS score > 80 (*P* = 0.0412), extent of resection (EOR) ≥ 90% (*P* = 0.0469), and Ki-67 index < 15% (*P* = 0.0025) were significantly associated with a longer OS (Fig. 2A–C). In contrast, chemoradiotherapy (*P* = 0.1749) and histological grade (*P* = 0.1682) did not reach statistical significance, whereas age > 55 years showed a trend toward poorer outcomes (*P* = 0.0660) (Fig. 2D–F).

**Fig. 3:**
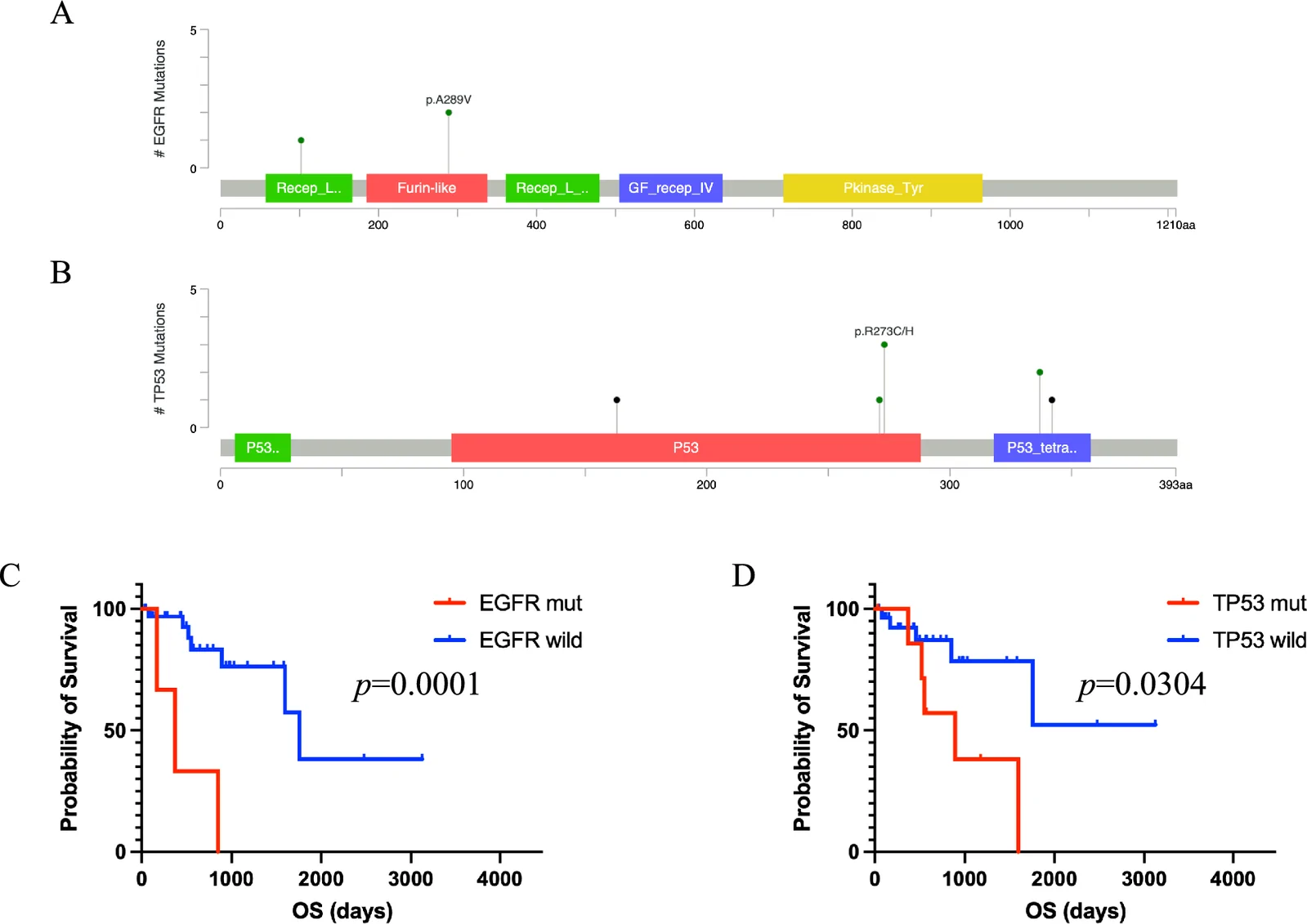
Association of somatic EGFR and TP53 mutations with overall survival in NEC. **A** *EGFR* domain structure showing somatic mutations, summarized using MutationMapper. **B** *TP53* domain structure showing somatic mutations, summarized using MutationMapper. Kaplan–Meier curves of overall survival stratified by **C** *EGFR* status and **D** *TP53* status. All *P*-values were derived from log-rank tests

In the NEC cohort, *EGFR* mutations were detected in three of 38 cases, all of which were located in the extracellular domain (ECD) of *EGFR* (Fig. 3A). Two carried the recurrent activating variant p.A289V, whereas the third, p.E102K, is a variant of uncertain significance located in ECD domain I (Fig. 3A). *TP53* mutations were identified in eight of 38 cases, with five mutations located in the p53 DNA-binding domain and three in the p53 tetramerization motif (Fig. 3B). Both *EGFR* and *TP53* mutations were significantly associated with poor OS in NEC (*P* = 0.0001 and *P* = 0.0304, respectively; Fig. 3C, D). *MGMT* promoter methylation was present in 11 of 30 NEC cases, and OS did not differ significantly between cases with and without *MGMT* promoter methylation (*P* = 0.6574, Supplementary Fig. 1).

### *EGFR* and *TP53* mutations define a high-risk subgroup within NEC

In univariate Firth-penalized Cox regression, both *EGFR* and *TP53* mutations were associated with markedly shorter overall survival (*EGFR*: HR 11.07, 95% CI 2.44–50.27, *P* = 0.003; *TP53*: HR 3.77, 95% CI 1.01–14.07, *P* = 0.040). To place these findings in the context of all prognostic factors, univariate hazard ratios for every clinical and molecular feature in Table 1, together with the combined *EGFR* and/or *TP53* group, are summarized in Supplementary Table 2; *EGFR* mutation, the combined *EGFR*/*TP53* group (HR 7.51, 95% CI 2.00–40.26, *P* = 0.002), extent of resection < 90%, Ki-67 ≥ 15%, and *TP53* mutation were each significantly associated with shorter survival, whereas the recurrent co-occurring alterations (*CDKN2A/B*, *NF1*, *PTEN*, *RB1*, and *PDGFRA*) were not. In PC1-adjusted multivariate Firth-penalized Cox regression, both alterations remained independent indicators of poor prognosis (*EGFR*: HR 1.61, 95% CI 1.02–2.57, *P* = 0.028; *TP53*: HR 1.55, 95% CI 1.00–2.42, *P* = 0.041); both estimates are summarized in Supplementary Table 2.

**Fig. 4:**
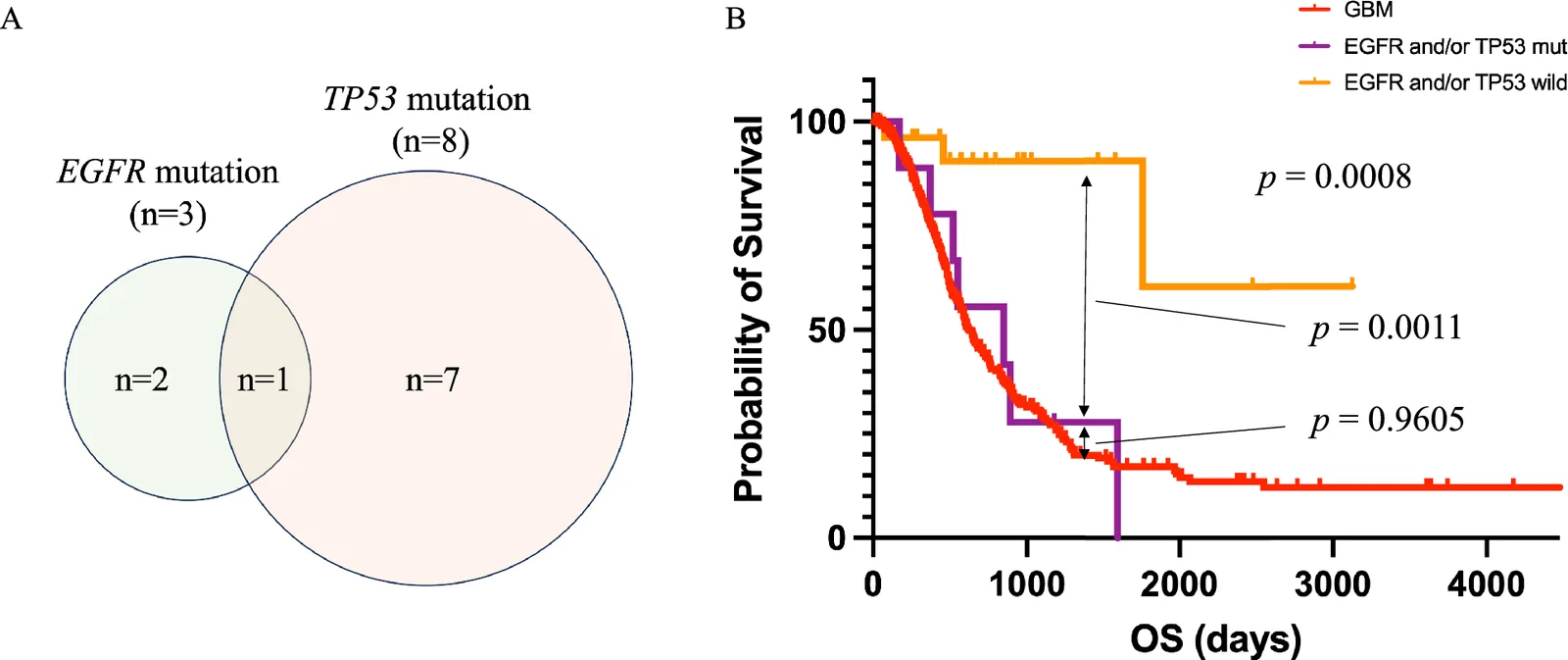
Combined EGFR and TP53 mutation status defines a high-risk subgroup of NEC. **A** Venn diagram showing the distribution and co-occurrence of *EGFR* and *TP53* alterations across the 38 NEC cases. **B** Kaplan–Meier curves of overall survival comparing GBM (n = 429), the *EGFR* and/or *TP53*-mutant group (n = 10), and the *EGFR* and *TP53*-wildtype group (n = 28). *P*-values were derived from log-rank tests

Because both *EGFR* and *TP53* mutations were individually associated with poor survival, we next examined whether the presence of either alteration could identify a high-risk NEC subgroup. The Venn diagram in Fig. 4A illustrates the overlap of *EGFR* and *TP53* mutations among the NEC cases. Seven tumors (18.4%) harbored a *TP53* mutation alone, two tumors (5.3%) harbored an *EGFR* mutation alone, and one tumor (2.6%) was double-positive (Fig. 4A). Based on this distribution, patients were stratified into three groups for survival analysis: NEC with *EGFR* and/or *TP53* mutations, NEC without either mutation, and the reference GBM cohort. The *EGFR* and/or *TP53*-mutant and *EGFR* and/or *TP53*-wildtype groups had broadly comparable clinical and genetic characteristics; however, EOR differed significantly between the groups (*P* = 0.038, Supplementary Table 1). Kaplan–Meier analysis revealed a significant OS difference among the three groups (*P* = 0.0008, Fig. 4B). Pairwise comparisons showed that NEC with *EGFR* and/or *TP53* mutations was associated with significantly shorter survival than NEC without either mutation (*P* = 0.0011, Fig. 4B), whereas no significant difference was observed between NEC with *EGFR* and/or *TP53* mutations and the GBM cohort (*P* = 0.9605, Fig. 4B). The survival curve of NEC with *EGFR* and/or *TP53* mutations closely paralleled that of GBM, indicating that these alterations identify a molecularly distinct NEC subgroup with a clinical course comparable to that of GBM.

We next assessed whether the survival findings could be confounded by differences in surgical sampling between the cohorts. The NEC cohort underwent less extensive surgery than the GBM cohort, with a higher proportion of biopsies (42.1% vs. 25.9%; *P* = 0.037) and fewer gross or ≥ 90% resections (26.3% vs. 46.9%; *P* = 0.017). Nevertheless, within NEC, overall survival did not differ between biopsied and resected cases (log-rank *P* = 0.80), and the *EGFR*/*TP53*-mutant and -wildtype subgroups were biopsied at similar rates (50% vs. 39%; *P* = 0.71). In a sensitivity analysis restricted to resected (non-biopsy) NEC cases (n = 22), *EGFR* and/or *TP53* mutations remained significantly associated with shorter overall survival (median 27.9 vs. 57.8 months; log-rank *P* = 0.003).

### Outcome of grade 2/3 tumors with an isolated *TERT* promoter mutation

We compared the overall survival of grade 2/3 *IDH*-wildtype tumors with isolated *TERT* promoter mutation, defined by the absence of *EGFR* amplification and + 7/ − 10 (n = 20), with that of histological grade 4 glioblastoma (n = 397) and NEC (n = 38). Survival of the isolated-*TERTp* group was significantly longer than that of histological grade 4 glioblastoma (median 42.9 vs. 19.6 months; log-rank *P* = 0.0019) but did not differ from that of NEC (median 52.5 months; *P* = 0.92; overall *P* < 0.0001) (Supplementary Fig. 2A). Within the isolated-*TERTp* group, *EGFR* and/or *TP53* mutation status was not significantly associated with overall survival (median 51.5 vs. 42.1 months; *P* = 0.9920), although the number of events was limited (Supplementary Fig. 2B).

### Methylation class family assignments and UMAP analysis

To further investigate the molecular identity of NEC and assess whether these tumors harbor a recognizable epigenetic signature, we performed genome-wide DNA methylation profiling in 30 of the 38 NEC cases with sufficient available DNA and analyzed the data using the DKFZ brain tumor methylation classifier and UMAP-based unsupervised dimensionality reduction.

Applying a calibrated score threshold of ≥ 0.9 for a confident methylation class family (MCF) assignment, most NEC cases failed to match any established reference class. As summarized in Table 2, 21 of the 30 tumors (70.0%) were classified as “no match,” indicating that their genome-wide methylation profiles did not correspond to any defined entity in the current reference cohort. Among the remaining nine cases, classifier calls were distributed across six different categories: three cases (10.0%) matched the MCF “control tissue”; two cases (6.7%) were assigned to the MCF “Glioblastoma, *IDH*-wildtype”; and one case each (3.3%) was classified as “Diffuse high-grade astrocytoma, MAPK pathway-altered,” “Gliomatosis cerebri-like glioma, *IDH*-wildtype,” “High-grade astrocytoma with piloid features,” and “Supratentorial pilocytic astrocytoma” (Table 2).

**Table 2:** Methylation class family assignments of NEC analyzed by genome-wide DNA methylation profiling

	Methylation class family	n
NEC (n = 30)	No match	21
	Methylation class family control tissues	3
	Methylation class family Glioblastoma, IDH-wildtype	2
	Methylation class family Diffuse high-grade astrocytoma, MAPK pathway-altered	1
	Methylation class family Gliomatosis Cerebri-like Glioma, IDH-wildtype	1
	Methylation class family High Grade Astrocytoma With Piloid Features	1
	Methylation class family Supratentorial Pilocytic Astrocytoma	1

**Fig. 5:**
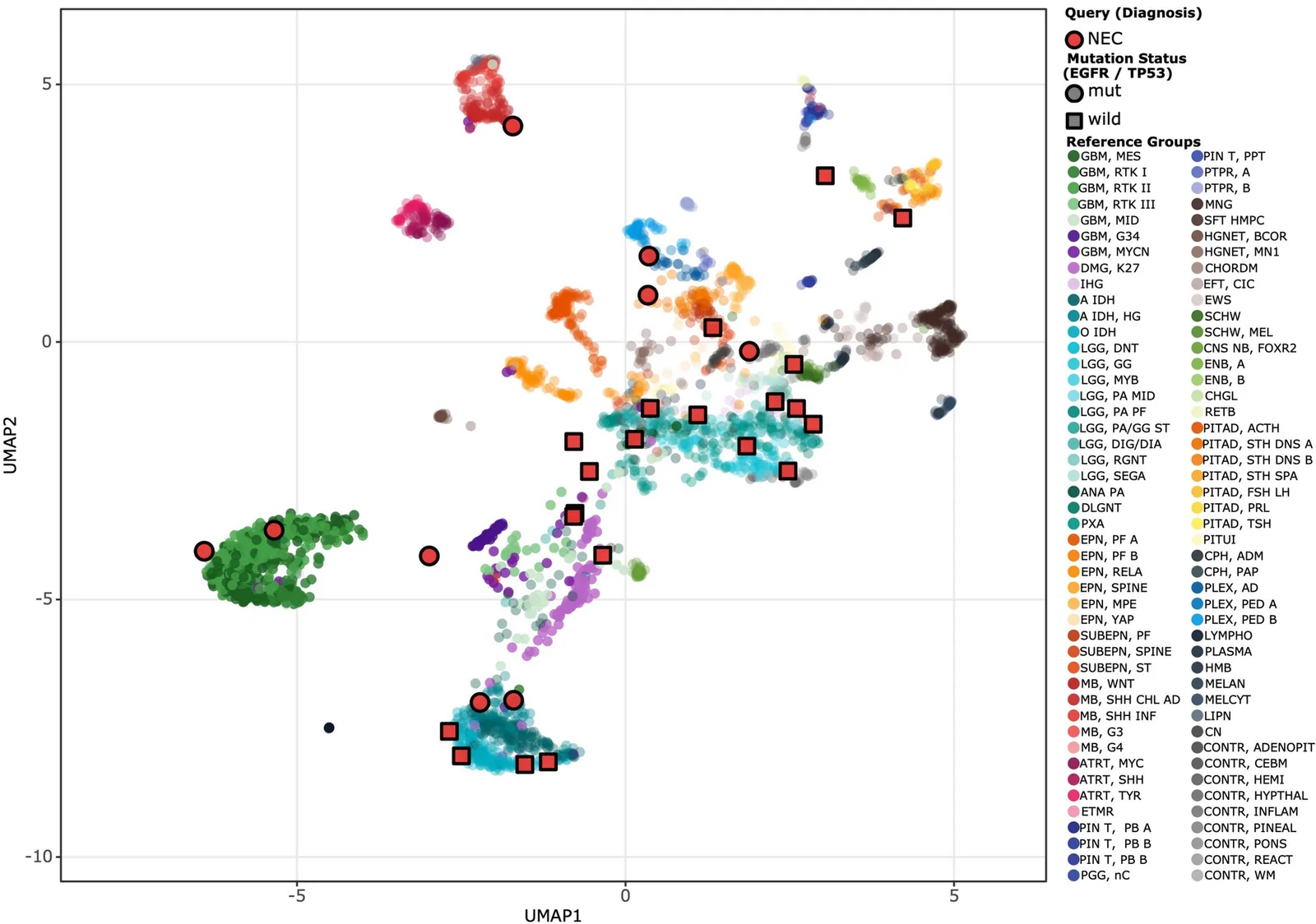
Methylation-based UMAP analysis of NEC. UMAP plot generated from the genome-wide DNA methylation data of 30 NEC cases jointly analyzed with 3,905 central nervous system tumor reference samples from NCBI GSE109381. NEC cases are shown in red, with EGFR and/or TP53-mutant cases indicated by circles and EGFR/TP53-wildtype cases indicated by squares

Unsupervised UMAP analysis incorporating NEC samples (red) together with reference cases representative of all major central nervous system tumor methylation classes corroborated these results (Fig. 5). Rather than forming a coherent, self-contained cluster, the 30 NEC cases were dispersed across the embedding space. Some tumors were localized adjacent to glioblastoma, *IDH*-wildtype, and other high-grade glial reference groups, whereas others mapped near low-grade glioma clusters, consistent with their classifier calls (Fig. 5). Notably, three NEC cases harboring *EGFR* or *TP53* mutations (circles in Fig. 5) were embedded in close proximity to the *IDH*-wildtype glioblastoma reference cluster. The remaining unclassifiable cases occupied intermediate or peripheral positions of the embedding, which were distant from any defined reference cluster (Fig. 5).

## Discussion

A central diagnostic challenge in adult-type *IDH*-wildtype astrocytomas is the residual category of “Not Elsewhere Classified” (NEC)—histological grade 2–3 tumors that fail to meet the WHO 2021 molecular criteria for GBM and consequently lack a defined prognostic anchor [12]. Although initially introduced as a placeholder for biologically uncertain cases, NEC has emerged as a heterogeneous group whose clinical behavior ranges from indolent, lower-grade disease to rapidly progressive courses indistinguishable from those of GBM [11, 13]. Molecular markers capable of resolving this heterogeneity have not yet been established, leaving clinicians without a consistent basis for risk stratification within this diagnostically grey zone.

In this multi-institutional cohort of 38 NEC cases, the established clinical variables—preoperative KPS, extent of resection, and Ki-67 labeling index—retained prognostic value consistent with previous reports on diffuse glioma outcomes [13, 34]. Beyond these clinical features, point mutations in *EGFR* or *TP53*, present in approximately one-quarter of the patients (10/38, 26.3%), independently predicted poor survival in Firth-penalized Cox regression. Patients harboring either alteration exhibited a survival curve closely parallel to that of GBM cohorts, whereas NEC patients lacking these mutations followed a markedly more favorable course. This dichotomy suggests that NEC is not a uniform clinical entity but a composite of at least two biologically distinct populations that can be distinguished through a simple two-gene assessment.

Notably, *EGFR* point mutations, rather than amplifications, define a part of this aggressive phenotype. NEC excludes cases harboring *EGFR* amplification because such tumors are reclassified as molecular GBM under the WHO 2021 framework [1]. The activating *EGFR* mutations detected in our NEC cohort were confined to ECD and therefore represent a biologically distinct class of oncogenic events that fall outside the current diagnostic criteria. ECD missense mutations—most prominently at A289 position—have been shown to confer ligand-independent, constitutive pathway activation, enhanced invasive growth, and resistance to standard therapies [20, 35]. While the present series identified only three *EGFR*-mutant cases, limiting the precision of effect estimates, the observation that all three mutations clustered in the ECD is consistent with the hypothesis that activating ECD mutations capture an oncogenic phenotype distinct from amplification. This distinction may also partly explain why *EGFR* amplification alone is insufficient to predict the benefit of *EGFR*-targeted therapies in GBM, as illustrated by prior *EGFR*-targeted approaches and a negative phase III trial of depatuxizumab mafodotin in *EGFR*-amplified newly diagnosed glioblastoma [36, 37]. Mechanistically, EGFR point mutations and EGFRvIII promote constitutive signaling and molecular reprogramming distinct from EGFR amplification, providing a biological rationale for their aggressive behavior [21–24]. These observations reinforce the interpretation that the extracellular-domain point mutations observed in our cohort, although rare, identify a biologically aggressive subset of NEC rather than incidental passenger events. We note, however, that the three ECD variants differ in their level of functional validation: two are recognized activating mutations, whereas the third (p.E102K) is a variant of uncertain significance in domain I, adjacent to the recurrently mutated R108 residue. We retained this case in the *EGFR*-mutant group because the variant was clonal—its allele frequency was comparable to that of a co-occurring *ATRX* alteration in the same tumor—and was predicted deleterious in silico, while explicitly acknowledging that its activating potential remains unproven.

*TP53* mutations carry independent prognostic significance within the NEC subgroup despite their classical association with *IDH*-mutant astrocytomas, where they typically co-occur with *ATRX* inactivation [28]. Their prognostic role in *IDH*-wildtype disease remains unclear [38]. The *TP53* mutations identified in our NEC cohort were enriched in the DNA-binding domain and tetramerization motif, with direct consequences for p53 function in maintaining genomic integrity, controlling apoptotic response, and modulating treatment sensitivity [27]. Within the *IDH*-wildtype NEC subgroup, our data indicate that loss of p53 function marks a biologically aggressive phenotype with survival comparable to that of GBM. The apparent discordance with *IDH*-mutant astrocytoma—in which *TP53* mutation carries little independent prognostic weight—may largely reflect differences in mutational frequency and clonal context. In *IDH*-mutant astrocytoma, *TP53* mutation is a near-ubiquitous, early clonal event that co-occurs with *ATRX* loss and is present in the great majority of tumors [28]; its near-universal prevalence leaves little contrast against which a survival difference could emerge. In *IDH*-wildtype NEC, by contrast, *TP53* mutation is confined to a minority of cases (8/38, 21.1%) and may therefore demarcate a biologically distinct, more aggressive subset. Consistent with a strong dependence on cohort composition, the prognostic value of *TP53* mutation in histologically defined *IDH*-wildtype glioblastoma has itself been inconsistent across studies [38].

Genome-wide DNA methylation profiling provides independent evidence of NEC molecular heterogeneity. Among the 30 NEC cases analyzed, 21 (70.0%) failed to match any reference methylation class family, and the remaining nine were distributed across six different categories, with no single class predominating. Unsupervised UMAP analysis recapitulated this pattern: NEC tumors did not form a coherent cluster with subsets localized near *IDH*-wildtype GBM reference groups, and individual cases mapped to lower-grade glioma clusters, consistent with their classifier calls. This high no-match rate is diagnostically informative, indicating that NEC, as currently defined, does not constitute a single epigenetic entity, but rather a residual collection of methylation phenotypes that the current reference cohort cannot resolve. Although the number of *EGFR*- or *TP53*-mutant cases was limited, some of these tumors were localized near GBM methylation reference groups, suggesting that the mutation status may partly reflect a broader shift toward GBM-like biology. Methylation profiling and point-mutation testing therefore appear complementary rather than redundant. Methylation analysis demonstrates that NEC is heterogeneous at the epigenome level, while targeted mutation analysis provides a practical, individual-patient stratifier that can be deployed in routine workflows where high-quality methylation data are not available. The high no-match rate in our cohort is notable in light of previous large-scale studies with methylation-based CNS tumor diagnostics, which emphasized both the diagnostic utility of the classifier and the occurrence of diagnostically challenging cases outside established reference classes [31, 39]. Because NEC is defined by the absence of mGBM-defining alterations rather than a shared molecular feature, its scattered UMAP distribution is consistent with a biologically heterogeneous residual category rather than a single epigenetic entity. This interpretation further supports the practical value of orthogonal, individual-patient markers such as *EGFR* and *TP53* point mutations, which can flag aggressive biology even in cases that cannot be resolved into any established methylation class.

Equally important is the corollary of this stratification: the majority of NEC cases—28 of 38 (73.7%) lacking both *EGFR* and *TP53* mutations—followed a markedly more favorable course than the GBM cohort. This sizeable favorable-outcome subgroup argues against collapsing NEC wholesale into the glioblastoma spectrum and supports the existence of a genuine, if uncommon, lower-grade *IDH*-wildtype astrocytoma whose biology and prognosis are distinct from those of GBM  [7, 16, 17]. Recognizing these patients carries practical weight, as their overtreatment under a uniformly GBM-like paradigm would be difficult to justify. A complementary priority for future work will therefore be to define the positive molecular features that characterize this indolent subgroup, rather than its defining absences, so that favorable-risk NEC can be identified prospectively rather than by exclusion alone.

Our findings should be interpreted in the context of a rapidly evolving literature on NEC and mGBM. The most directly comparable study was that of Cho et al., who recently reported the clinical and molecular characteristics of 46 patients with diffuse astrocytoma, *IDH*-wildtype, NEC, from a single institution [13]. In that cohort, multivariable analysis identified older age, higher Ki-67 index, and non-gross total resection as independent predictors of unfavorable OS, but specific gene-level alterations were not retained as independent prognostic factors. Our results are concordant with the clinical variables but diverge with respect to *EGFR* and *TP53* mutations, which were independently prognostic in our cohort. This could be attributed to differences in cohort composition (multi-institutional versus single-center), coverage of the targeted sequencing panel employed, and statistical methodology (Firth penalized Cox regression in our study, designed to handle rare events). Therefore, replication in independent cohorts is essential before considering a two-gene stratifier.

These observations align with cIMPACT-NOW Update 11 [14], which raised concerns about applying the current WHO 2021 mGBM-defining criteria in isolation—particularly in younger patients, in tumors with an isolated *TERT* promoter mutation, and in cases where *EGFR* amplification or + 7/ − 10 occurs outside its typical clinical or histological context. Broader convergent evidence for heterogeneity within the lower-grade *IDH*-wildtype and mGBM categories has been reported by Berzero et al. [7], Wijnenga et al. [4], and Priesterbach-Ackley et al. [6], all of which support the view that the current diagnostic criteria do not fully resolve the prognostic spectrum of these tumors. Update 11 indicates the need for refined criteria that integrate additional molecular features. Our data extend this perspective by showing that, within the residual NEC category, a minimal two-gene stratifier can flag biologically aggressive disease even when the WHO 2021 mGBM-defining alterations are absent. We do not propose redefining mGBM according to *EGFR* or *TP53* point mutations, given the small number of *EGFR*-mutant cases in our series; rather, we suggest that future iterations of the diagnostic framework consider these point mutations as candidate co-criteria for risk stratification in NEC to be evaluated prospectively in larger cohorts. The identification of NEC patients harboring these mutations may warrant closer radiographic surveillance and prioritization in molecularly matched clinical trials, mirroring the management considerations applied to GBM. Conversely, NEC patients lacking these alterations may be appropriate candidates for enrollment in lower-grade glioma protocols.

Consistent with the evolving evidence that isolated TERT promoter mutation alone may not confer a grade 4 clinical course [8–10], the grade 2/3 tumors in our cohort defined as glioblastoma solely by an isolated TERT promoter mutation followed a course indistinguishable from NEC rather than from histological glioblastoma. Incidentally, a similar reappraisal of the prognostic role of TERT promoter mutation has recently emerged in other tumor types, most notably meningioma [40, 41].

This study has some limitations that warrant considerations. First, its retrospective, multi-institutional design introduces the possibility of treatment-related confounding, and its long accrual window (2006–2025) spans multiple eras of standard-of-care evolution. Second, although the NEC cohort (n = 38) is among the larger such series reported, the small number of *EGFR*-mutant cases (n = 3) limits the precision of effect estimates and precludes finer subgroup analyses; further sensitivity analyses and external replication are needed to confirm the stability of the *EGFR*-related survival signal. Third, we did not apply formal multiple-testing correction to the gene-level univariate screen; therefore, the *EGFR* and *TP53* signals should be regarded as hypothesis-strengthening rather than definitive. Fourth, the NEC cohort underwent less extensive surgery than the GBM cohort, raising the possibility that focal histological grade-4 features were undersampled in some biopsied NEC cases. Several observations, however, argue against this explaining the *EGFR*/*TP53* association: the molecular glioblastoma-defining alterations were assessed by sequencing on the same material and are reliable irrespective of sample size; within NEC, biopsy status was not associated with survival; the poor-outcome *EGFR*/*TP53*-mutant cases were not preferentially biopsied; and the *EGFR*/*TP53* association persisted when the analysis was restricted to resected cases. Occult grade-4 histology in a minority of biopsied cases nonetheless cannot be entirely excluded. Finally, our findings have not yet been validated in an independent external cohort.

## Conclusions

*EGFR* and *TP53* point mutations identified a biologically coherent and clinically aggressive subset within *IDH*-wildtype astrocytomas currently classified as NEC, with outcomes comparable to those of GBM. This simple two-gene stratifier captures a molecular axis of malignancy that lies outside the current WHO 2021 GBM-defining alterations, and provides a practical framework that can be readily integrated into existing diagnostic workflows. Within the context of the call by cIMPACT-NOW Update 11 for the refinement of high-grade glioma criteria, our findings suggest that *EGFR* and *TP53* point mutations are candidate molecular features warranting evaluation in future iterations of the diagnostic framework for this otherwise residual category.

## Supplementary Information

Additional file1: Supplementary Fig. 1 Overall survival according to MGMT promoter methylation status. Kaplan–Meier curves comparing overall survival according to MGMT promoter methylation status in NEC. The *P*-value was derived from the log-rank test.

Additional file2: Supplementary Fig. 2 Overall survival of grade 2/3 IDH-wildtype tumors with an isolated TERT promoter mutation. Kaplan–Meier curves of overall survival for (A) histological grade 4 glioblastoma (n = 397), grade 2/3 tumors with isolated TERT promoter mutation, defined by the absence of EGFR amplification and +7/−10 (n = 20), and NEC (n = 38); and (B) the isolated-TERTp cohort stratified by EGFR and/or TP53 mutation status. All P-values were derived from the log-rank test. A B. A. C. B. *p* = 0.0412. *p* = 0.0025. *p* = 0.0469. D. E. F. p = 0.1682. *p* = 0.0660. *p* = 0.1749. A. B. C. D. *p* = 0.0001. *p* = 0.0304. A. B. *p* = 0.0008. *p* = 0.0011. *p* = 0.9605

Additional file3

Additional file4

## Data Availability

All data used and analyzed in the current study are available from the corresponding author upon reasonable request.

## Abbreviations

GBM: Glioblastoma
KPS: Karnofsky performance status
mGBM: molecular glioblastoma
NEC: Not elsewhere classified
OS: Overall survival
PCA: Principal component analysis
UMAP: Uniform manifold approximation and projection
WHO: World Health Organization
