# Supplementary material for: EGFR and TP53 mutations refine prognostic stratification of IDH-wildtype astrocytomas, not elsewhere classified
Source: Acta Neuropathol Commun. 2026 Aug 3;14:227. doi: 10.1186/s40478-026-02397-6 (PMC13721387; doi:10.1186/s40478-026-02397-6)
Supplement: Supplementary file 1 [file 40478_2026_2397_MOESM1_ESM.pdf]

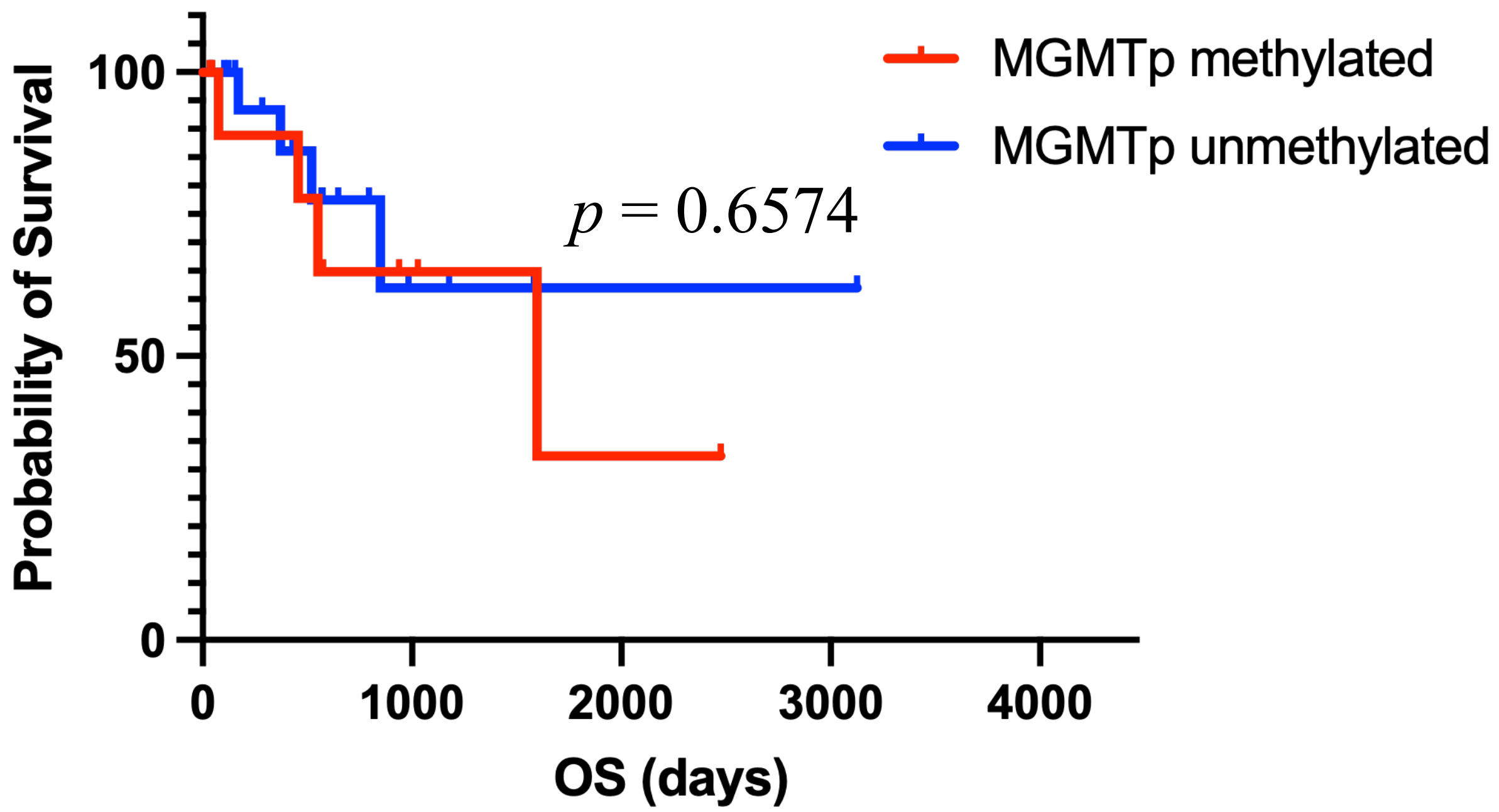

MGMTp methylated
MGMTp unmethylated
$p = 0.6574$
Probability of Survival
100
50
0
0
1000
2000
3000
4000
OS (days)
