# Supplementary material for: EGFR and TP53 mutations refine prognostic stratification of IDH-wildtype astrocytomas, not elsewhere classified
Source: Acta Neuropathol Commun. 2026 Aug 3;14:227. doi: 10.1186/s40478-026-02397-6 (PMC13721387; doi:10.1186/s40478-026-02397-6)
Supplement: Supplementary file 2 [file 40478_2026_2397_MOESM2_ESM.pdf]

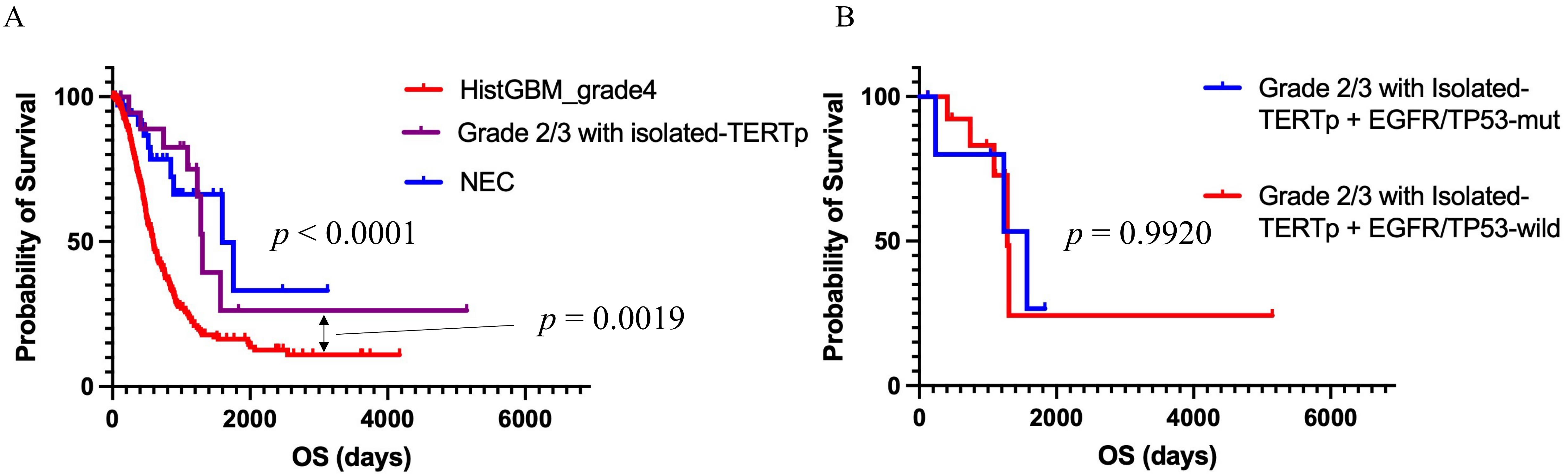
A
Probability of Survival
100
50
0
0
2000
4000
6000
OS (days)
HistGBM_grade4
Grade 2/3 with isolated-TERTp
NEC
p < 0.0001
p = 0.0019
B
Probability of Survival
100
50
0
0
2000
4000
6000
OS (days)
Grade 2/3 with Isolated-TERTp + EGFR/TP53-mut
Grade 2/3 with Isolated-TERTp + EGFR/TP53-wild
p = 0.9920
