# Supplementary material for: EGFR and TP53 mutations refine prognostic stratification of IDH-wildtype astrocytomas, not elsewhere classified
Source: Acta Neuropathol Commun. 2026 Aug 3;14:227. doi: 10.1186/s40478-026-02397-6 (PMC13721387; doi:10.1186/s40478-026-02397-6)
Supplement: Supplementary file 3 [file 40478_2026_2397_MOESM3_ESM.docx]

**Supplementary table. 1 Clinicopathological and molecular characteristics of patients stratified by *EGFR* and *TP53* mutation status.**

| Prognostic factor | | All (n=38) | EGFR and/or TP53 mut (n=10) | EGFR and/or TP53 wild (n=28) | *p*-value |
| --- | --- | --- | --- | --- | --- |
| Sex | male | 22 | 7 | 15 | 0.469 |
|  | female | 16 | 3 | 13 |  |
| Age | years | 59.2±14.9 | 63.6±12.8 | 57.6±15.5 | 0.284 |
| KPS score | >80 points | 24 | 6 | 18 | 1.000 |
|  | <80 points | 14 | 4 | 10 |  |
| Resection | ≥90 % | 10 | 0 | 10 | 0.038* |
|  | <90 % | 28 | 10 | 18 |  |
| Ki-67 (%) | | 17.8±17.3 | 24.3±22.6 | 15.4±14.7 | 0.169 |
| *CDKN2A/B* HD | | 11 | 3 | 8 | 1.000 |
| *PTEN* loss and/or mut | | 10 | 4 | 6 | 0.404 |
| *RB1* loss and/or mut | | 8 | 4 | 4 | 0.170 |
| *PDGFRA* alterations | | 11 | 3 | 8 | 1.000 |
| *NF1* loss and/or mut | | 10 | 3 | 7 | 1.000 |

KPS, Karnofsky Performance Status; mut, mutation; amp, amplification; HD, homozygous deletion.

*indicates statistical significance.
