# Supplementary material for: EGFR and TP53 mutations refine prognostic stratification of IDH-wildtype astrocytomas, not elsewhere classified
Source: Acta Neuropathol Commun. 2026 Aug 3;14:227. doi: 10.1186/s40478-026-02397-6 (PMC13721387; doi:10.1186/s40478-026-02397-6)
Supplement: Supplementary file 4 [file 40478_2026_2397_MOESM4_ESM.docx]

**Supplementary Table 2.** Firth-penalized Cox analysis of overall survival for every clinical and molecular factor in the NEC cohort

| **Factor** | **Univariate** | | **PC1-adjusted multivariate** | |
| --- | --- | --- | --- | --- |
|  | **HR (95% CI)** | **P** | **HR (95% CI)** | **P** |
| **Clinical / pathological features** | | | | |
| Age >55 y | 3.47 (0.90–19.07) | 0.072 | — | — |
| Male sex | 1.75 (0.44–9.57) | 0.438 | — | — |
| KPS <80 | 3.43 (1.00–11.76) | 0.050 | — | — |
| EOR <90% | 8.32 (1.07–>100) | 0.041 | — | — |
| Histological grade 3 | 2.36 (0.68–9.93) | 0.180 | — | — |
| Ki-67 ≥15% | 6.93 (1.82–37.54) | 0.004 | — | — |
| **Molecular alterations** | | | | |
| *CDKN2A/B* HD | 1.26 (0.30–4.32) | 0.726 | — | — |
| *NF1* loss/mutation | 1.97 (0.54–6.55) | 0.287 | — | — |
| *PTEN* loss/mutation | 1.57 (0.43–5.21) | 0.469 | — | — |
| *RB1* loss/mutation | 0.55 (0.10–2.01) | 0.384 | — | — |
| *PDGFRA* alteration | 1.61 (0.38–5.67) | 0.487 | — | — |
| *TP53* mutation | 3.77 (1.01–14.07) | 0.040 | 1.55 (1.00–2.42) † | 0.041 |
| *EGFR* mutation | 11.07 (2.44–50.27) | 0.003 | 1.61 (1.02–2.57) † | 0.028 |
| **Combined group** | | | | |
| *EGFR* and/or *TP53* mutation | 7.51 (2.00–40.26) | 0.002 | — | — |

CI, confidence interval; EOR, extent of resection; KPS, Karnofsky Performance Status; NEC, not elsewhere classified.

†Multivariate models include clinical PC1 (derived from principal component analysis of clinical variables) and gene variable. Clinical variables used for PCA: age, sex, histological grade, Ki-67, KPS, tumor location, treatment variables, and dissemination status. PCA was performed using centered and scaled variables; the first principal component (PC1) was used as a summary clinical covariate. Two-sided p-values are shown; no adjustment for multiple comparisons is applied in this table.
